# Pancan-meQTL: a database to systematically evaluate the effects of genetic variants on methylation in human cancer

**DOI:** 10.1093/nar/gky814

**Published:** 2018-09-07

**Authors:** Jing Gong, Hao Wan, Shufang Mei, Hang Ruan, Zhao Zhang, Chunjie Liu, An-Yuan Guo, Lixia Diao, Xiaoping Miao, Leng Han

**Affiliations:** 1 Department of Epidemiology and Biostatistics, Key Laboratory of Environmental Health of Ministry of Education, School of Public Health, Tongji Medical College, Huazhong University of Science and Technology, Wuhan, Hubei 430030, PR China; 2 Department of Biochemistry and Molecular Biology, The University of Texas Health Science Center at Houston McGovern Medical School, Houston, TX 77030, USA; 3 Department of Bioinformatics and Systems Biology, College of Life Science and Technology, Huazhong University of Science and Technology, Wuhan, Hubei 430074, PR China; 4 Department of Bioinformatics and Computational Biology, The University of Texas MD Anderson Cancer Center, Houston, TX 77030, USA

## Abstract

DNA methylation is an important epigenetic mechanism for regulating gene expression. Aberrant DNA methylation has been observed in various human diseases, including cancer. Single-nucleotide polymorphisms can contribute to tumor initiation, progression and prognosis by influencing DNA methylation, and DNA methylation quantitative trait loci (meQTL) have been identified in physiological and pathological contexts. However, no database has been developed to systematically analyze meQTLs across multiple cancer types. Here, we present Pancan-meQTL, a database to comprehensively provide meQTLs across 23 cancer types from The Cancer Genome Atlas by integrating genome-wide genotype and DNA methylation data. In total, we identified 8 028 964 *cis*-meQTLs and 965 050 *trans*-meQTLs. Among these, 23 432 meQTLs are associated with patient overall survival times. Furthermore, we identified 2 214 458 meQTLs that overlap with known loci identified through genome-wide association studies. Pancan-meQTL provides a user-friendly web interface (http://bioinfo.life.hust.edu.cn/Pancan-meQTL/) that is convenient for browsing, searching and downloading data of interest. This database is a valuable resource for investigating the roles of genetics and epigenetics in cancer.

## INTRODUCTION

The interpretation of the function of genomic variants, particularly in non-coding regions, is a major challenge for the genetic dissection of complex diseases such as cancer (1). Genome-wide association studies (GWAS) have identified numerous genetic loci that influence the risk of human cancer (2,3), but most of these loci are located in non-coding regions and are without clear molecular mechanisms that contribute to the phenotypic outcome. Previous studies considered a diverse set of functional regions, including miRNA binding sites, protein modification sites and transcription factor binding sites (4,5). However, the link between variants and epigenetic signals involved in the regulation of key biological processes has been largely overlooked.

As a major epigenetic mechanism that directs gene expression, DNA methylation plays a key role in the regulation of crucial biological and pathological processes (6). Aberrant DNA methylation is frequently observed in various cancers (7) and represents an attractive biomarker and therapeutic target (8,9). Increasing evidence indicates that single-nucleotide polymorphisms (SNPs) contribute to tumor initiation, progression and prognosis by influencing DNA methylation levels (10,11). Therefore, DNA methylation may be an important molecular-level phenotype that links a genotype with the trait of a complex disease. It is fundamentally vital to build a public data repository to identify SNPs that significantly affect DNA methylation levels, i.e. methylation quantitative trait loci (meQTL). Recent methodological advances allow for genome-wide screening of meQTLs in different tissues, including blood (12), lung (13) and brain (14). However, no database has been developed to systematically analyze meQTLs across multiple cancer types.

The Cancer Genome Atlas (TCGA) provides genome-wide genotype data and DNA methylation data for ∼10 000 cancer samples, making it possible to systematically conduct meQTL analysis across cancer types. We identified millions of meQTLs across 23 cancer types and have made them available for browsing, searching and downloading through Pancan-meQTL (http://bioinfo.life.hust.edu.cn/Pancan-meQTL/), a database for systematic evaluation of the effects of genetic variants on DNA methylation across diverse cancer types.

## DATA COLLECTION AND PROCESSING

### Genotype data collection, imputation and processing

**Figure 1.**
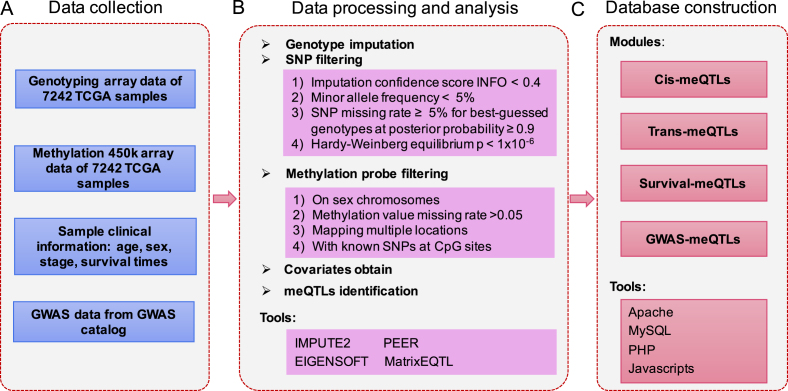
Data collection, processing and database construction. Pancan-meQTL collected the genotype, methylation and clinical data from TCGA to evaluate the effects of SNPs on methylation levels. For each cancer type, the data were processed using a series of filtering and quality control steps. Four datasets, *cis*-meQTLs, *trans*-meQTLs, survival-meQTLs and GWAS-meQTLs, are included in the database.

We downloaded genotype data (level 2) from TCGA data portal (https://portal.gdc.cancer.gov/) (Figure 1A). We kept 7735 samples with both genotype data and methylation data. We then combined colon adenocarcinoma (COAD) and rectum adenocarcinoma (READ) as colorectal cancer (CRC) (15) and removed cancer types with sample size <100 primary tumor samples. Thus, for further analysis, we had 7242 samples across 23 cancer types. We performed genotype imputation and filtering per cancer type as described in our previous study (16). After imputation and quality filtering, on average, 4 318 218 genotypes per cancer type were included in the meQTL analysis.

### Methylation data collection and processing

Methylation beta values (level 3) obtained from TCGA data portal (https://gdc-portal.nci.nih.gov/) were measured by the Illumina Infinium HumanMethylation450 BeadChip array, which contained 485 512 probes for each sample. Due to the specific nature of methylation patterns on sex chromosomes (17), we focused on autosomes. In each cancer type, probes were filtered by the following criteria: (i) methylation beta value missing rate > 0.05, (ii) mapping to multiple locations on the genome (18) and (iii) containing known SNP (1000 Genome Phase3 (19), MAF > 0.01) at CpG sites (20,21) (Figure 1B). On average, 369 244 high-quality methylation probes per cancer type were used for analyses. To minimize the effects of outliers on the regression scores, the values for each probe across samples per cancer type were transformed into a standard normal distribution based on rank (17,22,23).

### Covariates

To correct for known and unknown confounders and increase the sensitivity of our analyses, we included several covariates. The top five principal components calculated by smartpca in the EIGENSOFT program (24) were included to control for ethnicity differences. To remove hidden batch effects and other confounders in the methylation data, we used PEER software (25) to select the first 15 PEER factors from the methylation data as covariates. We included the common confounders of age, sex, and tumor stage as additional covariates (21,23,26).

### Identification of meQTLs

Identification of meQTL is straightforward that to test whether individuals carrying different genotypes show different methylation levels (17,20,27,28). For each cancer type, we performed linear regression with MatrixEQTL (29) to determine the effects of genetic variation on methylation levels. We calculated the pairwise associations between each SNP and CpG site. We used HumanMethylation450 BeadChip array annotation to extract the location (hg19) of methylation probes. We downloaded the SNP location (hg19) from dbSNP (https://www.ncbi.nlm.nih.gov/projects/SNP/). We defined meQTLs as SNPs with false discovery rates (FDRs) calculated by MatrixEQTL <0.05 and the absolute value of correlation coefficient (*r*) ≥0.3. If the SNP was within 1 Mb from the probe location (22), we denoted it as *cis*-meQTL; and if the SNP was beyond that point, we denoted it as *trans*-meQTL.

### Identification of survival-associated meQTLs

To prioritize promising meQTLs, we identified meQTLs that may be associated with patient survival times. We downloaded the clinical data, including patient overall survival times, from TCGA data portal (https://gdc-portal.nci.nih.gov/). For each meQTL, we used the log-rank test to examine the association between genotypes and patient overall survival times. We plotted Kaplan–Meier (KM) curves to show the survival times for individuals carrying different genotypes. We defined meQTLs with FDR <0.05 as survival-meQTLs.

### Identification of meQTLs in GWAS regions

Risk SNPs identified in GWAS studies were downloaded from GWAS catalog (http://www.ebi.ac.uk/gwas/) (2). GWAS linkage disequilibrium (LD) regions were extracted from SNAP (https://personal.broadinstitute.org/plin/snap/ldsearch.php) (30) with parameters (SNP data set: 1000 Genomes Pilot 1; LD *r*^2^ threshold: 0.5; population panel: CEU; distance limit: 500 kb). The meQTLs that overlapped with GWAS tagSNPs and LD SNPs were identified as GWAS-meQTLs.

### Enrichment analysis

To assess the enrichment of meQTLs in DNA regulatory elements and GWAS loci, we generated a control data set of non-meQTL SNPs with minor allele frequency (MAF) and distance matched to the set of meQTLs for each cancer type. We downloaded transcription factor binding sites (TFBSs) of related ENCODE cell lines from the UCSC genome browser, and extracted cancer-specific GWAS loci from GWAS catalog. Enrichment analyses of meQTLs were performed by two-tailed Fisher's exact test with the following 2 × 2 table: columns; meQTL SNPs and non-meQTL SNPs, rows; SNPs within and not within the TFBSs/GWAS loci.

## DATABASE CONTENT AND USAGE

### Samples in Pancan-meQTL

Pancan-meQTL included 23 cancer types with sample size ≥100 in TCGA, covering 7242 tumor samples. The sample size of each cancer type ranged from 103 in skin cutaneous melanoma (SKCM) to 664 in breast invasive carcinoma (BRCA) (Table 1). For the genotype data, we obtained a median of 4 487 756 SNPs for each cancer type after imputation and quality control, ranging from 2 721 411 for BRCA to 5 121 896 for acute myeloid leukemia (LAML). For the methylation data, there were, on average, 384 903 probes for each cancer type after probe filtering and quality control. The range was from 380 594 for CRC to 385 618 for testicular germ cell tumors (TGCT).

**Table 1.** Overview of samples and meQTLs in Pancan-meQTL

					*Cis*			*Trans*		
Cancer type	Disease full name	No. of samples	No. of methylation probes	No. of genotypes	Pairs	meProbes^a^	*cis*_meQTLs	Pairs	meProbes^a^	meQTLs
BLCA	Bladder urothelial carcinoma	405	384903	4182865	502774	8136	301392	216341	46165	51295
BRCA	Breast invasive carcinoma	664	384084	2721411	340881	7289	203391	47355	1804	31118
CESC	Cervical squamous cell carcinoma and endocervical adenocarcinoma	290	383425	4289322	531458	11284	318082	212025	48741	46185
CRC	Colon adenocarcinoma + Rectum adenocarcinoma	354	380594	3879590	555556	10781	316517	185943	40872	55402
ESCA	Esophageal carcinoma	173	382712	4357977	408020	15726	260629	21790	5427	13297
HNSC	Head and neck squamous cell carcinoma	501	385146	4245789	706915	10590	392427	100579	16599	51195
KIRC	Kidney renal clear cell carcinoma	306	384916	4487756	827668	14184	455287	598701	95277	82497
KIRP	Kidney renal papillary cell carcinoma	271	384786	4827856	830528	14986	486170	232041	52484	58017
LAML	Acute myeloid leukemia	122	385529	5121896	408043	10758	283314	22473	606	17201
LGG	Lower grade glioma	487	385198	4611830	789126	11092	454090	151792	24257	61737
LIHC	Liver hepatocellular carcinoma	367	383962	4152031	449509	9378	272962	127824	28610	46149
LUAD	Lung adenocarcinoma	448	384670	4343043	525439	8848	297745	110266	19790	52156
LUSC	Lung squamous cell carcinoma	359	385034	3676672	598237	10039	334937	191909	44240	60597
PAAD	Pancreatic adenocarcinoma	177	382517	4989296	903193	24432	513599	120777	41820	34727
PCPG	Pheochromocytoma and paraganglioma	175	385235	4701955	589160	19388	385289	34905	2175	23719
PRAD	Prostate adenocarcinoma	479	385067	4801718	1129044	16366	574577	188046	46160	66084
SARC	Sarcoma	257	382095	4083674	458278	14059	287992	64709	22198	24734
SKCM	Skin cutaneous melanoma	103	384364	4838338	200763	6689	150232	8399	399	6332
STAD	Stomach adenocarcinoma	367	383290	4265676	687004	10723	382962	77711	9612	49489
TGCT	Testicular germ cell tumors	148	385618	4807863	470576	14871	309018	36183	3791	25912
THCA	Thyroid carcinoma	498	385449	4842972	830377	11908	477575	248274	36261	72317
THYM	Thymoma	120	385609	4940146	562478	17015	352356	28228	1981	22009
UCEC	Uterine corpus endometrial carcinoma	171	385407	4961809	314774	14181	218421	17953	2911	12881

^a^meProbes: methylation probes regulated by meQTLs.

### meQTLs in Pancan-meQTL

Per cancer type, the associations between each SNP and methylation probe were analyzed for *cis*- and *trans*-meQTL mapping by linear regression with adjusted covariates. In the *cis*-meQTL analysis, we identified a total of 13 619 801 meQTL-CpG pairs at FDR < 0.05 and |*r*| ≥0.3 in 23 cancer types, which corresponded to a median *P*-value <4.34 × 10^−7^. We found a total of 8 028 964 *cis*-meQTLs across the cancer types, with a median of 318 082 meQTLs per cancer type, and a range from 150 232 in SKCM to 574 577 in prostate adenocarcinoma (PRAD) (Table 1). These meQTLs control DNA methylation at a median of 11 284 CpG sites per cancer type. On average, 35.1% *cis*-meQTLs are associated with multiple CpG sites. In *trans*-meQTL analysis, we identified 3 044 224 meQTL-CpG pairs at FDR < 0.05 and |*r*| ≥0.3 in 23 cancer types, which corresponded to a median of *P*-value < 1 × 10^−9^. There are 965 050 *trans*-meQTLs, with a median of 46 185 meQTLs per cancer type, and a range from 6332 in SKCM to 82 497 in kidney renal clear cell carcinoma (KIRC) (Table 1).

We further identified 23 432 meQTLs associated with patient overall survival times across different cancer types at FDR < 0.05. The number of survival-meQTLs ranged from 218 in BRCA to 11 212 in PRAD. We compared meQTL results to GWAS data and found 2 214 458 meQTLs that overlap with GWAS linkage disequilibrium regions of one or multiple traits. Enrichment analyses showed that meQTLs are significant enriched in most of TFBSs, such as CTCF, SIN3AK20 and NRSF (Supplementary Figure S1) and GWAS loci (Supplementary Table S1).

## WEB DESIGN AND INTERFACE

The database was built based on Apache, MySQL, PHP and Javascripts (Figure 1C). A user-friendly web interface is provided to facilitate searching, browsing and downloading.

### Querying meQTLs

**Figure 2.**
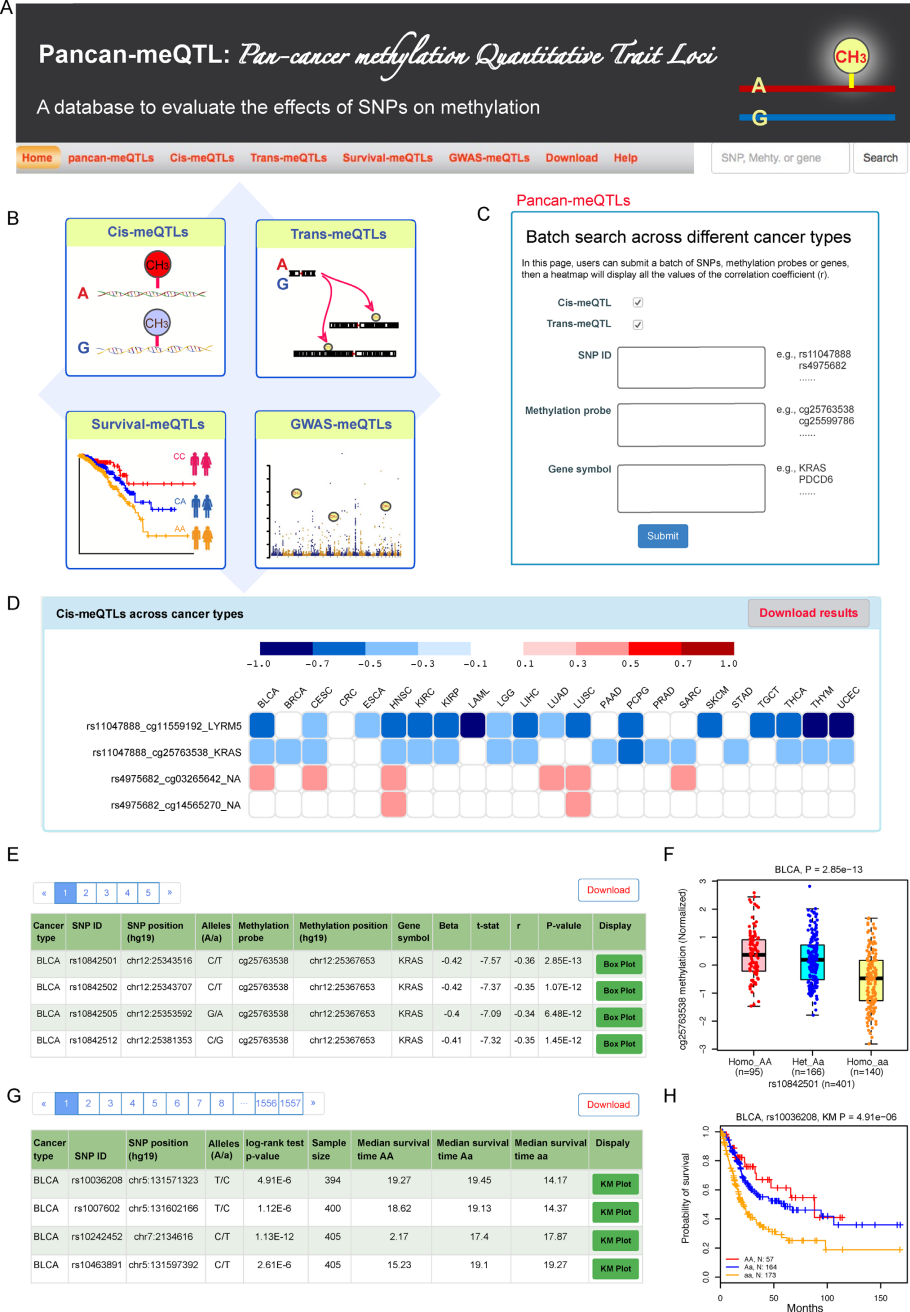
Overview of Pancan-meQTL database. (**A**) Browser bar, with a quick search box on the right. (**B**) Four modules in Pancan-meQTL: *cis*-meQTLs, *trans*-meQTLs, survival-associated meQTLs and GWAS-related meQTLs. (**C**) Batch search box allows users to input multiple SNPs, methylation probes and genes of interest. (**D**) Example of heatmap in pancan-meQTLs page showing the correlation coefficient across cancer types. (**E**) Records of *cis*-meQTLs on *cis*-meQTLs page. (**F**) Example of meQTL boxplot. (**G**) Records of survival-meQTLs on survival-meQTLs page. (**H**) Example of a Kaplan–Meier plot on survival-meQTL page.

We provide several entries for querying. Pancan-meQTL contains four datasets: *cis*-meQTLs, *trans*-meQTLs, survival-meQTLs and GWAS-meQTLs (Figure 2A and B). We developed four corresponding pages to display each dataset (Figure 2A). Users can enter each page from the browser bar to query SNPs, methylation data, and methylation-related genes of interest. On the ‘home’ page, we set a search section for users to query the four meQTL datasets. Also from the home page, users can search results by cancer type from the corresponding cancer type diagram. A quick search option is available on each page (top right) for searching by SNP ID, methylation probe or methylation-related gene symbol. Moreover, we provide a batch search page on the ‘Pancan-meQTLs’ page (Figure 2C). On this page, users can input multiple SNPs, methylation probes or genes of interest. A heatmap will show all the *r* values across all analyzed cancer types. For example, with inputs of ‘rs11047888’ and ‘rs4975682’, our results show that rs11044788 has significant correlations with cg11559192 and cg25763538 in 17 and 16 cancer types, respectively; whereas rs4975682 correlates with cg14565270 and cg03265642 in two and six cancer types, respectively (Figure 2D).

Querying on the *cis/trans*-meQTL page, a table with SNP ID, SNP genomic position, SNP alleles, methylation probe, methylation position, methylation-related gene symbol, gene position, beta value (effect size of SNP on gene expression), *r* value and *P*-value of meQTL will be returned (Figure 2E). For each record, a vector diagram of boxplot is provided to display the association between SNP genotypes and methylation levels (Figure 2F).

Querying on the survival-meQTL page, details with SNP ID, SNP genomic position, SNP alleles, log-rank test *P*-value and median survival times of different genotypes will be displayed (Figure 2G). A vector diagram of the KM plot is embedded in each record to display the association between SNP genotypes and overall survival times (Figure 2H).

Querying on the GWAS-meQTLs page will return the SNP information, related methylation, gene information and related GWAS traits. Search boxes are designed for retrieving specific cancer types, SNP, methylation probe and gene.

### Downloading data and figures

All the *cis/trans*-meQTLs for each cancer type can be downloaded from the ‘Download’ page. The queried results can be downloaded from the query page by clicking the ‘Download’ button. The *r* values of the batch search can be downloaded from the ‘Pancan-meQTL’ page. The vector diagrams of the boxplot and KM plot can be downloaded from the *cis/trans*-meQTL and survival-meQTL pages, respectively.

### Help section

The ‘Help’ page provides information for data collection, processing, result summary and contact. Pancan-meQTL welcomes any feedback by email to the address provided in the ‘Contact us’ section.

## CONCLUSION

We developed Pancan-meQTL as a resource that provides millions of meQTLs in multiple cancer types. Pancan-meQTL is the first public database that focuses on cancer-specific meQTLs. Our comprehensive analyses across 23 cancer types provide a great opportunity to investigate the patterns of meQTLs among cancer types. Among meQTLs, we also identified abundant meQTLs associated with patient survival time or located in known GWAS loci. These meQTLs are potentially promising candidates for genetic research. The Pancan-meQTL database will be continually updated as large-scale genotypic and methylation data become available. As a comprehensive database that characterizes meQTLs across multiple cancer types, Pancan-meQTL will be valuable for improving the interpretation of cancer risk-associated SNPs identified in genetic studies. It represents an important resource for cancer and epigenetic research.

## Supplementary Material

Supplementary Data
