# Supplementary material for: Pancan-meQTL: a database to systematically evaluate the effects of genetic variants on methylation in human cancer
Source: Nucleic Acids Res. 2018 Sep 7;47(Database issue):D1066–72. doi: 10.1093/nar/gky814 (PMC6323988; doi:10.1093/nar/gky814)
Supplement: Supplementary Data [file gky814_supplemental_files.zip › Supplementary_file_R1.docx]

**Supplementary figures**

**Supplementary Figure S1. Enrichment of meQTLs in transcription factor binding sites (TFBS) (x-axis) in multiple cancer types and related cancer cell lines (y-axis).** The green box represents analyzed TFs for specific cancer type. The size of inner box indicates the *P*-value of enrichment and the color of inner box represents the odds ratio (OR).

Supplementary Table S1. Enrichment analysis of meQTLs in GWAS loci

| Cancer type | Ratio of meQTL in GWAS (%) | Ratio of non-meQTL in GWAS (%) | Odds ratio | 95% CI^a^ | *P*-value |
| --- | --- | --- | --- | --- | --- |
| BRCA | 1.286 | 0.397 | 3.26 | 3.01-3.55 | 1.53$\times$10^-205^ |
| CRC | 0.366 | 0.143 | 2.56 | 2.28-2.88 | 1.97$\times$10^-64^ |
| LUAD | 0.540 | 0.151 | 3.59 | 3.20-4.02 | 6.19$\times$10^-133^ |
| LUSC | 0.541 | 0.155 | 3.51 | 3.16-3.90 | 2.85$\times$10^-150^ |
| PAAD | 0.045 | 0.051 | 0.89 | 0.73-1.09 | 0.274 |
| PRAD | 0.328 | 0.093 | 3.53 | 3.18-3.93 | 4.02$\times$10^-149^ |
| SKCM | 0.132 | 0.070 | 1.88 | 1.44-2.48 | 1.20$\times$10^-6^ |

^a^ CI: confidence interval
