# Supplementary figures and images for: Pancan-meQTL: a database to systematically evaluate the effects of genetic variants on methylation in human cancer
Source: Nucleic Acids Res. 2018 Sep 7;47(Database issue):D1066–72. doi: 10.1093/nar/gky814 (PMC6323988; doi:10.1093/nar/gky814)

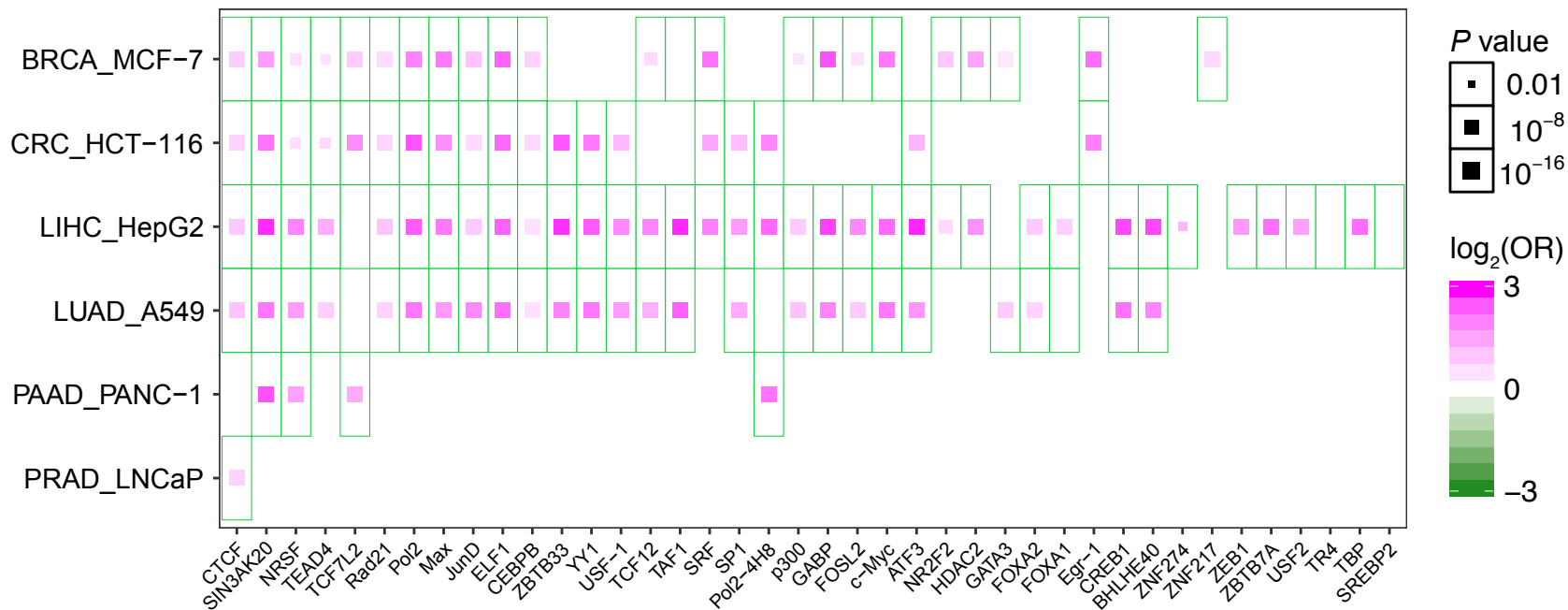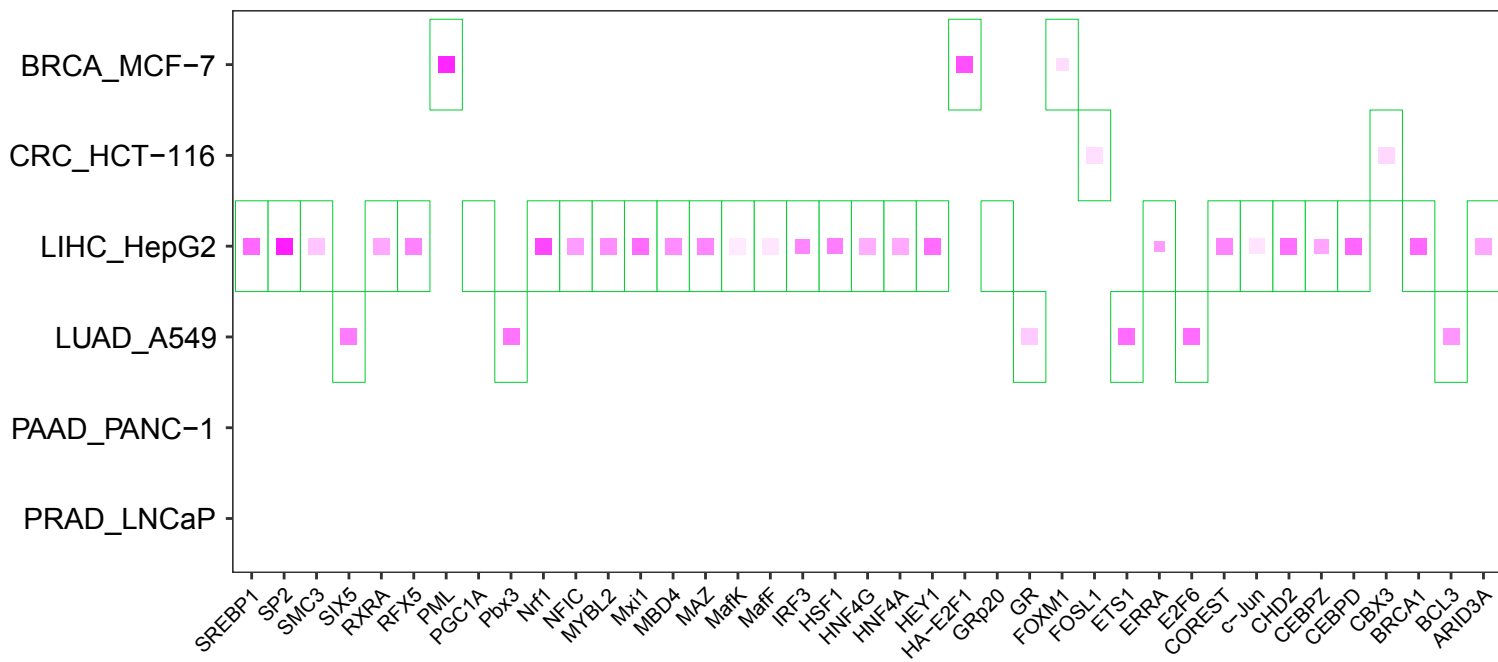

Supplement: Supplementary Data [file gky814_supplemental_files.zip › supp figure 1.pdf]
